# Eating Disorders: Prevalence of Anorexia Nervosa Among Tunisian Military Nursing Students

**DOI:** 10.1192/j.eurpsy.2025.1390

**Published:** 2025-08-26

**Authors:** Y. Ben Othmene, S. Bejaoui, W. Kabtni, A. Baatout, S. Eddif, I. Gafsi, H. Kefi, A. Oumaya

**Affiliations:** 1 Psychiatry, Military Hospital of Instruction, Tunis, Tunisia

## Abstract

**Introduction:**

The societal pressure to maintain a thin appearance combined with the academic stress has a significant influence on eating disorders in young people.

Anorexia Nervosa (AN), a severe eating disorder characterized by a distorted body image, restrictive eating habits, and an overwhelming fear of gaining weight, is often misdiagnosed.

Early detection is critical to improve treatment management.

**Objectives:**

The present study aimed to determine the prevalence of Anorexia Nervosa among Tunisian military nursing students.

**Methods:**

A cross-sectional descriptive survey was conducted from March to May 2024 on nursing students at the Military Health School of Tunisia, using a data file for data collection and an Arab version of a self-report questionnaire: Eating Attitudes Test (EAT40), validated in Tunisia. The EAT40 is designed to assess attitudes related to eating and body image serving also as a tool to identify Anorexia Nervosa. To analyze the obtained data, Excel software was used.

**Results:**

The study enrolled 148 students, mostly male (57.4%), with an average age of 21.3 [19-24] years. Of them, 48.6% were in their third year and 19.6% in their second one. Geographically, 57.4% was from the north of Tunisia. The majority (85.8%) was in a middling socioeconomic position and 9.5% was in a high one.

With an average weight of 75.1±14.8kg and extremes between 48kg and 105kg, 52.7% of the population weighed less than 70kg. Also, 67.56% of them were taller than 1m70 of whom 35.81% stood between 1m70 and 1m80. The average Body Mass Index was 23 [17-32]. Among the students, 71.6% had a normal BMI, whereas 2.7% were underweight.

Regarding outward look, 89.9% of respondents said they were happy with their bodies’ looks and 67.6% with their weight. When they were younger, the majority (73.6%) did not experience any weight issues. Prior to completing the EAT40, 57.4% said they had no eating disorders.

Of the surveyed, 83.3% expressed dissatisfaction with school meals for a variety of reasons, the most common being insufficient quantity, poor quality, or tasteless food.

According to the study, the average of the EAT40 score was 20±14. Of the participants, 10.8% (N=16) had Anorexia Nervosa; their ETA score was 30 or higher. The majority (62.5%,N=10), were female; females’ EAT40 average score was 23±15 [1-44] while males’ EAT40 average score was 21±10 [1-41].

The results also revealed that students suffering from AN had an average BMI of 19±4 [weight: 60±10kg, height: 1m60±.08m], compared to students without eating disorders with a BMI of 25±3 [weight: 69±12kg, height: 1m70±0.15m].

**Conclusions:**

This study reveals a concerning prevalence of Anorexia Nevrosa among Tunisian military nursing students, highlighting the necessity of early detection for effective management and of interventions such as raising awareness within educational institutions aspiring to better mental health outcomes for healthcare students.

**Disclosure of Interest:**

None Declared

